# Surveillance to improve physical activity of children and adolescents

**DOI:** 10.2471/BLT.22.288569

**Published:** 2022-10-31

**Authors:** John J Reilly, Salome Aubert, Javier Brazo-Sayavera, Yang Liu, Jonathan Y Cagas, Mark S Tremblay

**Affiliations:** a School of Psychological Sciences & Health, University of Strathclyde, 50 George Street, Glasgow, G1 1XQ, Scotland.; b Active Healthy Kids Global Alliance, Ottawa, Canada.; c Department of Sports and Computer Science, Universidad Pablo de Olavide, Seville, Spain.; d School of Physical Education, Shanghai University of Sport, Shanghai, China.; e Department of Sports Science, University of the Philippines Diliman, Quezon City, Philippines.; f Healthy Active Living and Obesity Research Group, CHEO Research Institute, Ottawa, Canada.

## Abstract

The global transition to current low levels of habitual physical activity among children and adolescents began in the second half of the last century. Low physical activity harms health in both the short term (during childhood and adolescence) and long term (during adulthood). In turn, low physical activity could limit progress towards several sustainable development goals, undermine noncommunicable disease prevention, delay physical and mental health recovery from the coronavirus disease 2019 pandemic, increase health-care costs and hinder responses to climate change. However, despite the importance of physical activity, public health surveillance among children and adolescents is very limited globally and low levels of physical activity in children is not on the public health agenda in many countries, irrespective of their level of economic development. This article details proposals for improvements in global public health surveillance of physical activity from birth to adolescence based on recent systematic reviews, international collaborations and World Health Organization guidelines and strategies. Empirical examples from several countries illustrate how improved surveillance of physical activity can lead to public health initiatives. Moreover, better surveillance raises awareness of the extent of physical inactivity, thereby making an invisible problem visible, and can lead to greater capacity in physical activity policy and practice. The time has arrived for a step change towards more systematic physical activity surveillance from infancy onwards that could help inform and inspire changes in public health policy and practice globally.

## Résumé

Durant la seconde moitié du siècle dernier, l'activité physique des enfants et adolescents a commencé à baisser à l'échelle mondiale pour atteindre le faible niveau actuel. La sédentarité a des effets néfastes sur la santé, tant à court terme (pendant l'enfance et l'adolescence) qu'à long terme (à l'âge adulte). Elle peut également entraver la progression vers de nombreux objectifs de développement durable, compromettre les efforts de prévention des maladies non transmissibles, retarder la guérison physique et mentale liée à la pandémie de maladie à coronavirus 2019, accroître les dépenses en soins de santé et freiner les réactions au changement climatique. Pourtant, malgré l'importance que revêt l'activité physique, rares sont les dispositifs de surveillance de la santé publique mis en place pour les enfants et adolescents dans le monde. En outre, la lutte contre la sédentarité chez les plus jeunes ne figure pas parmi les priorités de santé publique dans la plupart des pays, quel que soit le niveau de développement économique de ces derniers. Le présent article détaille une série de propositions visant à améliorer la surveillance de la santé publique en matière d'activité physique de la naissance à l'adolescence, fondées sur des revues systématiques récentes, des collaborations internationales ainsi que des stratégies et lignes directrices de l'Organisation mondiale de la Santé. Des exemples empiriques provenant de plusieurs pays illustrent comment cette amélioration peut déboucher sur des initiatives de santé publique. De plus, une meilleure surveillance permet de sensibiliser à l'ampleur de cette inactivité, donnant ainsi de la visibilité à un problème jusqu'alors invisible, et peut renforcer les capacités politiques et pratiques relatives à l'activité physique. Il est temps de procéder à un changement en profondeur afin de surveiller plus systématiquement l'activité physique dès le plus jeune âge, en vue d'orienter et d'encourager l'évolution des politiques et pratiques en la matière dans le monde.

## Resumen

La transición mundial hacia los niveles bajos actuales de actividad física regular entre niños y adolescentes comenzó en la segunda mitad del siglo pasado. La disminución de la actividad física perjudica la salud tanto a corto plazo (durante la infancia y la adolescencia) como a largo plazo (durante la edad adulta). A su vez, la falta de actividad física podría limitar el progreso hacia varios objetivos de desarrollo sostenible, socavar la prevención de enfermedades no transmisibles, retrasar la recuperación de la salud física y mental tras la pandemia de la enfermedad por coronavirus de 2019, aumentar los costes de la atención sanitaria y dificultar las respuestas al cambio climático. No obstante, a pesar de la importancia de la actividad física, la vigilancia de la salud pública entre los niños y los adolescentes es muy limitada a nivel mundial y los niveles bajos de actividad física en los niños no se incluyen en la agenda de salud pública de muchos países, sea cual sea su nivel de desarrollo económico. En este artículo, se detallan propuestas para mejorar la vigilancia de la actividad física en la salud pública a nivel mundial, desde el nacimiento hasta la adolescencia, a partir de recientes revisiones sistemáticas, colaboraciones internacionales y directrices y estrategias de la Organización Mundial de la Salud. Ejemplos empíricos de varios países ilustran cómo una mejor vigilancia de la actividad física puede conducir a iniciativas de salud pública. Asimismo, una mejor vigilancia aumenta la concienciación sobre el alcance de la inactividad física, haciendo evidente un problema poco visible, y puede conducir a una mayor capacidad en la política y la práctica de la actividad física. Ha llegado el momento de dar un paso adelante hacia una vigilancia más sistemática de la actividad física desde la infancia que podría ayudar a informar e inspirar cambios en la política y la práctica de la salud pública en todo el mundo.

## ملخص

بدأ الانتقال العالمي إلى المستويات المنخفضة الحالية، من النشاط البدني المعتاد بين الأطفال والمراهقين، في النصف الثاني من القرن الماضي. يؤدي انخفاض النشاط البدني للإضرار بالصحة على المدى القصير (أثناء الطفولة والمراهقة) وعلى المدى الطويل (خلال مرحلة البلوغ). وفي المقابل، يمكن أن يحد النشاط البدني المنخفض من التقدم نحو العديد من أهداف التنمية المستدامة، ويقوض الوقاية من الأمراض غير المعدية، ويؤخر تعافي الصحة البدنية والعقلية الناجمة عن جائحة مرض فيروس كورونا 2019، ويزيد من تكاليف الرعاية الصحية، ويعيق الاستجابات لتغير المناخ. ومع ذلك، فعلى الرغم من أهمية النشاط البدني، فإن مراقبة الصحة العامة بين الأطفال والمراهقين محدودة للغاية على الصعيد العالمي، والمستويات المنخفضة من النشاط البدني لدى الأطفال ليست مدرجة في جدول أعمال الصحة العامة في العديد من الدول، بغض النظر عن مستوى النمو الاقتصادي لديهم. تتناول هذه المقالة بالتفصيل مقترحات لتحسين مراقبة الصحة العامة العالمية للنشاط البدني منذ الولادة وحتى مرحلة المراهقة، بناءً على المراجعات المنهجية الحديثة، وحالات التعاون الدولي، وإرشادات واستراتيجيات منظمة الصحة العالمية. توضح الأمثلة التجريبية من العديد من الدول: كيف يمكن أن يؤدي تحسين مراقبة النشاط البدني إلى مبادرات للصحة العامة. وعلاوة على ذلك، يؤدي تحسين المراقبة إلى زيادة الوعي بمدى الخمول البدني، وبالتالي يحول المشكلة غير المرئية إلى مرئية، ويمكن أن يؤدي إلى قدرة أكبر في النشاط البدني من حيث السياسة والممارسة. لقد حان الوقت لتغيير الخطوة نحو المزيد من المراقبة المنهجية للنشاط البدني منذ الطفولة فصاعدًا، والتي يمكن أن تساعد في توعية وإلهام التغييرات في سياسة الصحة العامة وممارساتها على الصعيد العالمي.

## 摘要

从上世纪下半叶开始，全球范围内的儿童和青少年习惯性身体活动水平逐渐向目前的低水平转变。较低水平的身体活动会对健康造成短期（儿童和青少年时期）和长期（成年时期）危害。反之，较低水平的身体活动会限制实现多项可持续发展目标方面的进展、影响非传染性疾病的预防、延缓从 2019 年新型冠状病毒肺炎疫情中的身心健康恢复，增加健康护理成本并妨碍气候变化应对措施的实施。然而，尽管身体活动如此重要，全球范围内对儿童和青少年的公共健康监测却非常有限。在许多国家，不论其经济发展水平如何，较低的儿童身体活动水平仍未纳入公共健康议程。本文基于近期开展的系统性评审、国际合作项目和世界卫生组织指南和策略，详细介绍了对从出生到青少年时期身体活动的全球公共健康监测的改进建议。几个国家的实证案例表明，改进身体活动监测可促进公共健康行动的实施。此外，更好的监测还会提高人们对身体活动内容的认识，从而使隐藏的问题显现出来，并提高制定和实践身体活动政策的能力。现在是时候迈出一步了，从婴儿期开始进行更系统的身体活动监测，这可能有助于为全球公共卫生政策和实践的变革提供信息和启发。

## Резюме

Глобальный переход к нынешним низким уровням привычной физической активности среди детей и подростков начался во второй половине прошлого века. Низкая физическая активность вредит здоровью как в краткосрочной (в детском и подростковом возрасте), так и в долгосрочной перспективе (в зрелом возрасте). В свою очередь, низкая физическая активность может препятствовать достижению ряда целей в области устойчивого развития, отрицательно сказаться на профилактике неинфекционных заболеваний, замедлить восстановление физического и психического здоровья после пандемии коронавирусной инфекции 2019 года, увеличить расходы на здравоохранение и затруднить реагирование на изменение климата. Однако, несмотря на важность физической активности, надзор за состоянием здоровья детей и подростков в мире очень слабо развит, а низкий уровень физической активности у детей не стоит на повестке дня здравоохранения во многих странах, независимо от уровня их экономического развития. В этой статье подробно изложены предложения по улучшению глобального надзора в области общественного здравоохранения за физической активностью от рождения до подросткового возраста на основе последних систематических обзоров, международного сотрудничества и руководящих принципов и стратегий Всемирной организации здравоохранения. Примеры эмпирических данных нескольких стран показывают, как улучшение надзора за физической активностью может способствовать реализации инициатив в области общественного здравоохранения. Кроме того, более эффективный надзор повышает осведомленность о масштабах физической инертности, тем самым обнаруживая невидимую проблему, и может привести к повышению потенциала политики и практики в области физической активности. Пришло время сделать шаг вперед в направлении более систематического надзора за физической активностью с младенчества и далее, что может способствовать информированию и вдохновить на изменения в политике и практике общественного здравоохранения во всем мире.

## Introduction

The World Health Organization (WHO) recognizes that physical activity in childhood is essential for healthy development, for short- and long-term physical and mental health, for the achievement of several sustainable development goals (SDGs) and for the prevention of many noncommunicable diseases.^1^^–^^4^ The time spent on physical activity and on related behaviours (e.g. sedentary behaviour, including screen time and sleep) has a major impact on an individual’s health across their entire life-course from infancy and early childhood onwards.^4^ However, physical activity levels among children and adolescents globally are typically lower than recommended by evidence-based guidelines, even in low- and middle-income countries.^5^^–^^8^ This important deficit is hidden or distorted by a lack of public health surveillance of physical activity among children and adolescents. In many countries, surveillance is incomplete, intermittent or even absent, which means that low physical activity among children and adolescents is effectively invisible and, consequently, does not feature on the public health agenda.^6^^–^^8^

Here, we discuss the need for better global surveillance of physical activity among infants, children and adolescents in the light of recent systematic reviews, evidence-based guidelines and international surveillance collaborations. We argue that “what gets measured gets done” and “if you don’t measure, it you can’t change it.” Improved surveillance is essential for good public health policy and practice.^9^ Moreover, surveillance is recognized as a core public health activity for identifying the need for new policies, for monitoring trends, for identifying inequities and for evaluating the impact of policies. However, the failure to recognize the low physical activity among children and adolescents means that physical activity surveillance is often an undervalued and underfunded public health activity.^9^^,^^10^ For low- and middle-income countries, physical activity surveillance is particularly challenging given their limited capacity in what is a relatively new area of public health,^10^ their limited resources and the coexistence of other major public health issues, such as undernutrition, infectious disease and child labour.^11^ In this paper, we report recent examples from around the globe which show that improved surveillance of physical activity can and does stimulate physical activity policy and practice. We also argue that a renewed emphasis on promoting physical activity among children and adolescents is timely given the current global public health agenda, which includes: (i) the physical activity target for adolescents in WHO’s global action plan on physical activity 2018–2030;^1^ (ii) the role of physical activity in tackling the global noncommunicable disease crisis, in achieving several SDGs and in responding to the climate emergency;^3^ and (iii) the need to recover from the adverse impact of the coronavirus disease 2019 (COVID-19) pandemic on physical activity, on other behaviours (e.g. on screen time and time spent outdoors) and on related health outcomes (e.g. obesity, mental health and physical fitness).^7^^,^^12^^,^^13^

## Low physical activity globally

In the past decade, international studies and surveillance initiatives have found that typical physical activity levels among school-age children and adolescents globally are much lower than recommended, even in low- and middle-income countries.^5^^–^^8^ In October 2022, the Global Matrix 4.0 surveillance study was published by the Active Healthy Kids Global Alliance.^6^ The Global Matrix initiative is an international collaboration that provides up-to-date, comprehensive and global assessments of: (i) levels of physical activity and related behaviours and health indicators (e.g. sedentary behaviour and physical fitness) in school-age children and adolescents; and (ii) factors that influence those behaviours, such as government policy and practice, the local community and environment, schools, family and peer groups. The Global Matrix 4.0 study found that, consistent with previous studies, physical activity levels were typically far lower than recommended by WHO across low-, middle-, and high-income countries:^2^^,^^6^ no more than around one third of children and adolescents globally met the new recommendations. Moreover, few low- or middle-income countries had physical activity policies, whereas policy was well-developed in many high-income countries but implementation was often very limited. In addition, the Global Matrix 4.0 surveillance study also found multiple gaps and limitations in the public health surveillance of physical activity among children and adolescents around the world: for many countries (more often low- and middle-income countries than high-income countries), insufficient data were available to assess all 10 indicators of health behaviours and influences on health behaviours covered by Global Matrix surveys, which made it difficult to fully interpret the implications of the survey results.^6^

## Causes of low physical activity

**Fig. 1:**
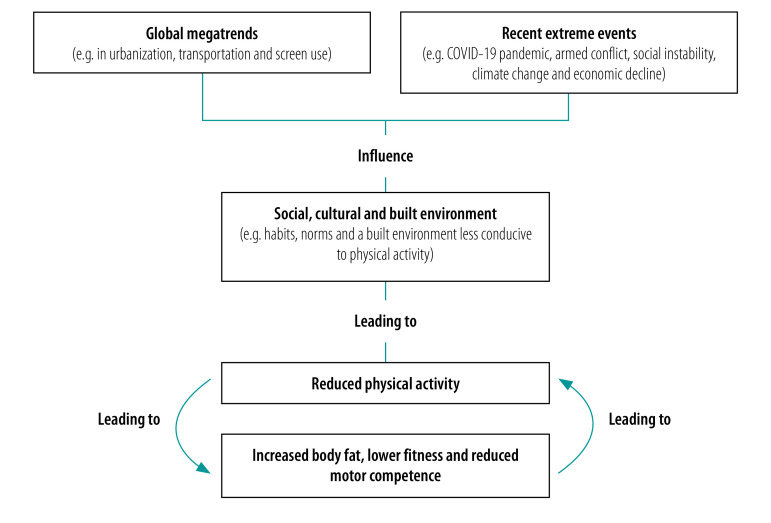
Factors contributing to reduced physical activity among children and adolescents

Children and adolescents were much more physically active in the distant past.^14^^,^^15^ Physical activity started to decline globally in the late 20th century and a so-called physical activity transition occurred, even in low- and middle-income countries.^16^ Levels of physical activity are influenced largely by socioecological factors rather than factors associated directly with health or physical activity itself.^6^^,^^9^^,^^10^ The physical activity transition among children and adolescents, as among adults, was mainly due to so-called global megatrends, such as increased urbanization, reduced active transportation (e.g. walking and cycling) and increased screen time associated with greater access to television, the internet and mobile devices, which displaced time previously spent in physical activity (Fig. 1).^17^ The rapid and widespread nature of the physical activity transition supports the view that its causes were neither local nor national but were universal and environmental and analogous to the causes of the obesity pandemic, which occurred around the same time.^18^ Contemporary sociocultural and physical environments globally are generally not conducive to high levels of habitual physical activity among children and adolescents.^6^ Instead, they have produced abnormal activity habits and social norms, have limited access to basic biological needs and denied a human right: the right to physically active play.^19^ Like obesity, low physical activity among children and adolescents can be viewed as a normal response to an abnormal environment. Against the backdrop of an unsupportive environment for physical activity, the Active Healthy Kids Global Alliance Global Matrix 4.0 surveillance study found,^6^ across the globe, that very recent, and in some cases relatively new, extreme environmental changes were associated with acute declines in opportunities for physical activity among school-aged children. These included: (i) climate change, which can cause extreme heat, wildfires and air pollution, thereby reducing access to outdoor physical activity, and which can cause a loss of snow and ice at higher latitudes, thereby reducing opportunities for winter play and sports; (ii) COVID-19 restrictions, such as school closures; (iii) economic decline, which reduces opportunities for activity outside the home; and (iv) conflicts within and between nations, which can reduce the perceived safety of the outdoor environment, thereby limiting opportunities for physical activity among children displaced from their homes.

COVID-19: coronavirus disease 2019.

Note: The global megatrends and extreme events that can reduce child and adolescent physical activity may also lead to biological changes that reduce physical activity even further.

The environmental changes that have reduced physical activity among children and adolescents have also led to biological changes, including reduced motor competence (i.e. impaired fundamental movement skills), reduced physical fitness and high body fat, even among those who are not overweight or obese as defined by their body mass index. In turn, these biological changes further reduce physical activity by producing feedback loops that amplify adverse environmental impacts on physical activity (Fig. 1).^20^^–^^25^

## Limitations of surveillance

The main limitations of existing global surveillance programmes of physical activity among children and adolescents are summarized in Table 1. These include the underrepresentation, or even absence, of surveillance in low- and middle-income countries, in children younger than 4 years and in children aged 5 to 9.9 years,^7^ despite physical activity levels being low in many of these countries and the decline beginning in middle childhood.^5^^,^^6^^,^^26^ Although the idea that moderate- or vigorous-intensity physical activity declines markedly at puberty and with the onset of adolescence is well established in public health, it is a myth.^26^ Marked inequalities in physical activity are often established well before adolescence in many countries.^6^^,^^27^ However, the national surveillance programmes needed to identify these inequalities are frequently absent or inadequate – sample sizes may be too small or the samples may not be representative. In the absence of systematic surveillance, inequalities will not usually be visible and will, therefore, not be targeted by physical activity policy or interventions.^7^^,^^9^ Moreover, particular groups of children have been underrepresented or actively excluded from public health surveillance of physical activity, including: (i) those with a chronic disease or disability;^28^ (ii) those who do not attend school, which is especially common in low- and middle-income countries; (iii) those who live in rural settings, who still comprise the majority in many low- and middle-income countries; and (iv) those belonging to indigenous populations. It is also difficult to survey the physical activity associated with child labour, which is often ignored and clearly unethical. Moreover, the consequences of low physical activity for healthy child development are poorly understood but are likely to be negative.

**Table 1:** Limitations of current physical activity surveillance programmes for children and adolescents globally,^a^ 2022

Surveillance programme limitation	Relevance	Specific problems with current programmes^b^	Solution
Low- and middle-income countries are underrepresented	The so-called physical activity transition means that low physical activity is now common, even in low- and middle-income countries	Not global; validity of method uncertain; surveys intermittent; or surveys not representative (e.g. urban bias)	Conduct truly global surveys with proven reliability and validity after translation into local languages
Some population groups are underrepresented, absent or actively excluded, such as children (especially those younger than 5 years), individuals with a chronic disease or disability, individuals living in a rural setting and children not at school	WHO guidelines and strategies apply to individuals from birth onwards, including those with a chronic disease or disability and children not at school, who are numerous and at a high risk of low physical activity	The SUNRISE programme was limited to children aged 3–4 years	Conduct more-inclusive surveys covering individuals from birth to 18 years of age that could be linked to comprehensive national health surveys and demographic and health surveys
Surveys are irregular	Assessing changes over time is important because changes may influence policy evaluations and capacity-building	Irregular (e.g. most surveillance is opportunistic); or not financially sustainable	Conduct global surveys at more frequent intervals (e.g. every 5 years)
Few data are available on inequalities associated with, for example, gender, socioeconomic status, age, disability or urban or rural residence	Substantial inequalities exist and may be getting worse	Data coverage of subgroups is limited	Conduct surveys large enough to identify and report on inequalities between population subgroups
Measurement methods lack validity, reliability or cultural appropriateness	There is a need for new research on the validity, reliability and cultural appropriateness of surveillance programmes	The SUNRISE programme uses valid and culturally appropriate methods but includes only children aged 3–4 years	Conduct a global, methodological study to establish the validity, reliability and cultural appropriateness of surveillance measures
Limited range of variables measured and limited surveillance of 24-hour movement behaviours	Few data are available on sedentary behaviour and sleep; and WHO recommendations on physical activity, sedentary behaviour and sleep are limited to children younger than 5 years	Not all programmes include a wide range of variables	Expand future surveys to cover a more comprehensive range of variables
No surveillance of behaviours that influence physical activity (e.g. the environment and public health policy)	Knowledge of higher-level influences on physical activity is crucial; and socioecological models of health require the assessment of influences on behaviour	Only the AHKGA-GM programme attempts to capture influences on physical activity at different levels of a socioecological model	Expand future surveillance programmes to cover behavioural influences
Surveillance not in accordance with WHO guidelines	As guidelines on moderate-to-vigorous-intensity physical activity have evolved, surveillance can be left behind (e.g. the change from a minimum of 60 min/day of moderate-to-vigorous-intensity physical activity every day to an average of 60 min/day)	The AHKGA-GM programme is based on WHO guidelines but does not use common measures	Ensure measurement methods (e.g. device-based measures) are flexible enough to adapt to new evidence and new guidelines
Physical activity dose not measured in different domains (e.g. sport, active play, active transportation, physical education, chores and work or occupational activity) and quality of physical activity not recorded (e.g. type of sport practised or mode of active transportation)	Meeting WHO guidelines on physical activity requires accumulating activity in multiple domains; and knowledge of the location, timing and nature of physical activities is essential for developing interventions	Although indicators of physical activity are evaluated in most AHKGA-GM surveys, neither their dose (i.e. duration, intensity and frequency) nor their context (e.g. during sport, active play, active transportation, physical education, chores or occupational activity) is usually assessed	Develop a global, harmonized, adaptable, methodological approach to the measurement of time spent in each activity domain
Heterogeneity of surveillance methods between and within surveys	Use of different surveillance methods hampers comparability	The varied surveillance methods used in different AHKGA-GM surveys make comparisons between countries challenging and often invalid	Develop standardized and harmonized global surveys that use valid, reliable and culturally appropriate methods and that are adaptable to the local geographical and cultural context. This will require the use of accelerometry or of simpler methods validated against accelerometry

AHKGA-GM: Active Healthy Kids Global Alliance Global Matrix; WHO: World Health Organization.

^a^ Individuals younger than 10 years are classified as children and those aged 10.0 to 19.9 years are classified as adolescents.

^b^ Current programmes include: (i) the AHKGA-GM programme, which is global, covers children aged 5–17 years, involves the use of devices and questionnaires, is carried out every 2–3 years and includes measures of environmental influences on physical activity; (ii) the Global School-Based Student Health Survey (GSHS), which is global and opportunistic, covers children aged 13–17 years and is questionnaire-based; (iii) the Health Behaviours in School-aged Children (HBSC) programme, which is conducted in Europe and North America, covers adolescents aged 11–15 years, is carried out typically every 4 years and is questionnaire-based; (iv) the International Children’s Accelerometry Database (ICAD), which is global and opportunistic, covers children aged 3–18 years and is device-based; (v) the International Study of Childhood Obesity, Lifestyle and the Environment (ISCOLE) programme, which was global, covered children aged 10 years, included the surveillance of environmental influences, was device-based and ended in 2016; and (vi) the SUNRISE international surveillance study of 24-hour movement behaviours in the early years, which is global (with an emphasis on low- and middle-income countries), covers children aged 3–4 years, is opportunistic and involves the use of devices and questionnaires.

The validity, reliability and cultural appropriateness of the methods used to measure physical activity in many surveys are not supported by good-quality evidence and the variation in methods used by different surveys renders it difficult, or even impossible, to make international comparisons (Table 1).^7^ In addition, the limitations and gaps in physical activity surveillance common across countries at all levels of economic development can delay, prevent or misinform policy (Box 1).^29^^–^^36^ National and global surveillance programmes rarely assess the dose, domain, antecedents or determinants of physical activity: (i) the physical activity dose includes the duration, frequency and intensity of each physical activity; (ii) the physical activity domain could be active transportation, active play, physical education, organized sport, organized physical activity, chores and, potentially, child labour; and (iii) the antecedents or determinants of physical activity include the family, peers, school, community facilities and programmes, the built and social environment, and public health policy.^6^^,^^7^ A lack of policy implementation and evaluation remains the norm across the globe, even in countries that have a good written physical activity policy or strategy (Box 1).^6^ Furthermore, many countries do not have physical activity policies targeting physical activity in children or policies on sedentary behaviour or screen time for children or adolescents.^6^^,^^37^

Box 1 **Effect of surveillance limitations on public health policy and practice around child and adolescent physical activity, China, Philippines and Scotland, 2008–2022**

**China**

In China, an upper-middle-income country, gaps in physical activity surveillance of children and adolescents and in physical activity policy implementation and evaluation were reported between 2017 and 2022.^29^^,^^30^

Beginning in 2007, the Chinese government has released a series of policies and strategies to promote physical activity among children and adolescents.^29^ However, there has been a lack of policy implementation and no systematic national surveillance of child or adolescent physical activity has been conducted. Consequently, the impact of changes in policy and practice could not be assessed with confidence.^30^

**Philippines**

In the Philippines, a lower-middle-income country where adolescent physical activity is very low, no surveillance of physical activity in children was conducted between 2018 and 2022, there was limited capacity for physical activity surveillance and little physical activity policy was either established or implemented.^31^^–^^33^

As in many low- and middle-income countries, surveillance of child and adolescent physical activity in the Philippines has been restricted to adolescents, as reported in the country’s global school-based student health surveys.^31^ These surveys indicate that typical physical activity levels among adolescents were low and have been further declining recently: the percentage of adolescents with a sufficient level of physical activity was 14% in 2011 and 7% in 2019.^32^ To date, physical activity policies have been directed mainly at the school environment, with a particular emphasis on physical education. Policy implementation has been slow and evaluation has been almost non-existent. Moreover, very little policy on sedentary behaviour was reported in 2022.^32^ A situation analysis of children in the Philippines conducted by the United Nations Children's Fund in 2018 stated that, “policies and laws have remained largely ‘on paper’ due to insufficient efforts to put the necessary systems, financing, and capacity in place for implementation.”^33^

**Scotland**

In Scotland, physical activity policy and practice between 2008 and 2022 has been misinformed by inaccurate measurement of physical activity during surveillance.^34^^–^^36^

The Scottish physical activity strategy is well-developed and has been guided by surveillance evidence derived largely from nationally representative Scottish Health Surveys.^34^ However, these surveys used an invalid physical activity questionnaire that vastly overestimated moderate-to-vigorous physical activity and, erroneously, found high levels of moderate-to-vigorous-intensity physical activity during childhood and a very marked decline in moderate-to-vigorous-intensity physical activity during adolescence, particularly among girls.^35^^,^^36^ This spurious finding provided the rationale for directing Scottish policy and practice towards adolescent girls for many years as they were the perceived high-risk group for low physical activity in the population.^36^ In fact, physical activity in both boys and girls was reported to be low by early childhood over a decade ago.^34^^,^^35^ Consequently, there is a need for population-wide actions that begin much earlier in the life-course, that include both boys and girls and that extend to other major areas of concern not included in current policy, such as sedentary behaviour.

**Fig. 2:**
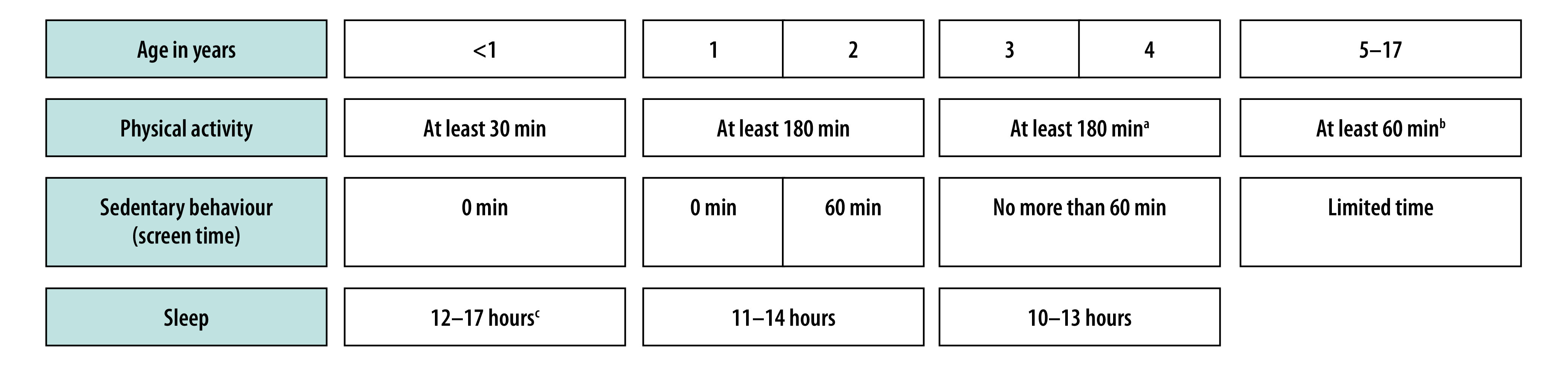
WHO guidelines on daily physical activity, sedentary behaviour and sleep for infants, children and adolescents^2^^,^^4^

For children younger than 4 years and preadolescents, the limitations of self- and proxy-reporting of physical activity mean that device-based measurement is an essential component of surveillance.^9^^,^^38^ The SUNRISE international surveillance study of 24-hour movement behaviours in the early years,^38^ which includes both rural and urban populations in low- and middle-income countries, found that partnerships between developed and developing countries can lead to the creation of a combination of device-based measures and questionnaires for conducting the surveillance of physical activity, sedentary behaviour and sleep in children aged 3 to 4 years in accordance with WHO guidelines (Fig. 2). In addition, the SUNRISE study’s findings indicate that only a minority of children aged 3 to 4 years satisfy these guidelines, even in rural areas and in low- and middle-income countries.^38^ In recent years, the new paradigm of assessing 24-hour movement behaviours (i.e. integrating measures of physical activity, sedentary behaviour and sleep) has been incorporated into WHO guidelines for children younger than 5 years and into some national guidelines for all infants, children and adolescents.^39^ However, surveillance of sedentary behaviour and sleep is even more limited internationally than surveillance of physical activity and, hence, substantial improvement is required (Table 1**)**.^40^

WHO: World Health Organization.

^a^ It is recommended that 60 of the 180 min of activity are of moderate or vigorous intensity.

^b^ It is recommended that all 60 min of activity are of moderate or vigorous intensity.

^c^ It is recommended that infants younger than 3 months sleep for 14 to 17 hours and those aged 4 to 12 months sleep for 12 to 16 hours.

Notes: The time spent asleep was considered beyond the scope of WHO’s 2020 guidelines for school-age children and adolescents, and the guideline development group concluded that there was insufficient evidence to specify a limit for time spent in sedentary behaviour (including screen time).

## Grounds for optimism

The global megatrends (Fig. 1) that have contributed to reduced physical activity among children and adolescents are probably reversible to only to a limited degree, yet there are grounds for optimism and positive developments have been reported, even in resource-poor settings. In the Philippines, for example, concern about the effect of COVID-19 restrictions on physical activity recently led to the successful implementation of policies promoting cycle lanes.^41^ In addition, although average physical activity levels are low in high-income countries, subgroups of 15 to 20% of children (mainly boys) have been reported to maintain high levels of moderate- or vigorous-intensity physical activity throughout their entire childhood and adolescence,^26^^,^^42^^,^^43^ even in such an unfavourable physical and cultural environment. They have avoided the declines in moderate-to-vigorous-intensity physical activity that begin in other children around school entry age. Moreover, the negative biological feedback loops that help drive the extent of low physical activity (Fig. 1) can potentially be broken by directly increasing physical activity. In 2020, WHO modified its evidence-based guidelines on the time spent by school-age children and adolescents in moderate-to-vigorous-intensity physical activity from at least 60 minutes per day every day to an average of at least 60 minutes per day (Fig. 2).^2^ This change is likely to produce a modest increase in the proportion of children and adolescents who satisfy the guidelines.

Guidance for public health policy-makers and practitioners on tackling low physical activity among children and adolescents is available from WHO and is summarized in our key points for policy-makers and practitioners in Box 2.^1^^,^^2^^,^^4^ In its development and implementation, physical activity policy is beginning to include previously neglected groups, such as children and adolescents with disabilities.^47^ New policy development should also consider other groups that have been neglected to date, such as children and adolescents who work, who do not attend school or who live in especially vulnerable situations (e.g. as refugees). Additionally, recent research has found that effective physical activity policy can help countries meet several SDGs and that the benefit of increased physical activity extends beyond the physical activity domain itself.^3^ Greater understanding of these findings should help increase resources for physical activity policy and practice globally.

Box 2 **Key points for public health policy-makers and practitioners on tackling low physical activity among children and adolescents**

**Increasing physical activity**

- Increasing physical activity is more complex and challenging than it seems.
- Increasing physical activity requires informed action on its upstream environmental influences, such as transportation, the built environment, planning, the cultural environment and the regulation of screen time – these lie outside the domain of physical activity itself (Fig. 1).
- Focusing only on downstream individual policy targets (e.g. through educating the population) is unlikely to be very effective and may increase inequalities.
- Increasing physical activity meaningfully by targeting the most obvious individual physical activity domains (e.g. school and sport) will require informed and effective action in these domains, not just single policy targets. For example, targeting physical education alone or the school environment alone is unlikely to increase physical activity meaningfully because children are typically at school for only around half of all days annually and are generally less active on non-school days.^44^
- Meaningful increases in physical activity will require changes beyond the single domain of sport because physical activity does not occur in sport alone.
- Physical activity is very hard to measure accurately in children and adolescents. Self-reports and parental proxy reports of physical activity should be treated with caution unless there is good evidence of validity, reliability and cultural appropriateness.
- In addition to physical activity, policy and action are required on sedentary behaviour (e.g. screen time) and sleep.

**Guidance and support**

- Extensive guidance on physical activity policy and strategy, support and resources are available but could be used much more widely.
- WHO has produced many practical, evidence-based strategies and guidelines, such as the *Global action plan on physical activity 2018–2030* and *Guidelines on physical activity, sedentary behaviour and sleep for children under 5 years of age*.^1^^,^^4^ These documents are supported by a wide range of resources to help guide effective implementation of policies and strategies.
- WHO strategies and other initiatives suggest useful actions that can be taken to establish informed policy and practice, though these actions may need to be adapted to particular circumstances and may require dedicated resources.
- Developing policy, even informed policy, is insufficient. Successful policy requires implementation and evaluation, which are often lacking globally.^6^
- At present, policy-makers have limited awareness of the key public health concepts in child and adolescent physical activity and of the evidence available.
- International collaborations that focus on surveillance (e.g. the SUNRISE study, the Active Healthy Kids Global Alliance, the European FitBack collaboration and WHO’s STEPwise approach to noncommunicable disease surveillance, or STEPS)^6^^,^^38^^,^^45^^,^^46^ can provide support for physical activity policy, can help build surveillance capacity and can enable resources and findings to be shared between developed and developing countries.
- Existing surveillance data on physical activity in children and adolescents, though limited, are useful for guiding policy and practice. Making national survey data available as soon as possible would be helpful: in many low- and middle-income countries, global school-based student health survey data only become available after many years and are, therefore, much less useful than they could be.

**Risk groups**

- Assuming there are specific children or adolescents who are at a particularly high risk of insufficient physical activity is usually wrong. A group should only be labelled high-risk when there is robust surveillance evidence that the risk is higher than in the general population of children and adolescents.
- All children and adolescents are now at a high risk of insufficient physical activity at all ages from infancy onwards and in all places, including rural areas and low- and middle-income countries.
- Adolescence does not cause a decline in physical activity – physical activity declines well before adolescence and the decline continues throughout adolescence.

**Rights and benefits**

- Children have a fundamental right to physically active play – environments that do not support physical activity deny them that right.
- The benefits of physical activity for children and adolescents extend well beyond the substantial health effects and include, for example, educational and cognitive benefits.^2^
- Increased physical activity in children and adolescents also benefits society: it contributes to the achievement of several SDGs, aids noncommunicable disease prevention, helps counter the climate emergency and assists recovery from the COVID-19 pandemic.

COVID-19: coronavirus disease 2019; SDGs: sustainable development goals; WHO: World Health Organization.

## International partnerships

Physical activity in children and adolescents is a relatively new academic discipline and, at present, capacity is largely concentrated in developed countries.^10^ One major barrier to effective surveillance is limited capacity in the discipline, particularly in low- and middle-income countries. There is even less capacity in an area of movement behaviours that has only recently been recognized as important, namely sedentary behaviour and sleep. In the ongoing SUNRISE study in children aged 3 to 4 years,^38^ in WHO’s STEPwise approach to noncommunicable disease surveillance (STEPS) in adults and in the Active Healthy Kids Global Alliance Global Matrix initiative in school-age children and adolescents,^6^^,^^45^ global physical activity surveillance has involved partnerships between developed and developing countries. As a result, surveillance capacity has been built globally and awareness of low physical activity and high sedentary behaviour has been raised. In addition, these studies and initiatives have started to inform public health policy and action.^48^^,^^49^

The development of national report cards on physical activity among children and adolescents by the Active Healthy Kids Global Alliance has made a substantial contribution to physical activity surveillance and to public health policy and practice.^48^^,^^49^ The national report cards provide details of performance on six health behaviours and outcomes (i.e. overall physical activity, organized sport and physical activity, active play, active transportation, sedentary behaviours and physical fitness) and details of four influences on those health behaviours and outcomes (i.e. family and peers, school, community and environment, and government). In several countries, improved surveillance has led to major changes in public health policy.^48^^,^^49^ In Slovenia, for example, robust annual surveillance data detailed in the country’s national report cards demonstrated that physical fitness among children and adolescents was low and declining. This troubling finding resulted in a national policy on the design and implementation of sport and exercise clubs to be run during school time and after school across the country.^50^ Subsequent surveillance conducted using the national report card showed that this change in policy and practice was effective in improving child and adolescent physical fitness in the years after the change but before the COVID-19 pandemic.^50^

Several European nations are now developing, or have recently developed, school-based physical fitness surveillance programmes that involve relatively simple, low-cost measures. These nations have formed an international collaboration, called FitBack, that aims to build capacity in fitness surveillance and to share examples of how best to change policy and practice to maintain or improve child and adolescent fitness.^46^ This is one more example of how an international collaboration can use surveillance to make the invisible visible (in this case, low child and adolescent physical fitness) and to inspire and inform changes in public health policy and practice.

## Conclusions

Increasing physical activity and combating sedentary behaviour among children and adolescents globally will require a step change in surveillance. However, this step should not be simply a monitoring exercise. Our experience, supported by many international examples, suggests that improved surveillance will also inform and inspire changes in public health policy and practice. The time has arrived for making such a step change because: (i) physical activity is now recognized as important globally for achieving several SDGs and for preventing many noncommunicable diseases; (ii) WHO has produced new global guidelines on physical activity from infancy to adulthood;^2^^,^^4^ and (iii) there is a need to solve the physical activity crisis after the COVID-19 pandemic. Moreover, not monitoring physical activity adequately across the population’s life-course has substantial opportunity costs.
